# Noncompaction of the left ventricle: a new cardiomyopathy is presented to the clinician

**DOI:** 10.1590/S1516-31802006000100007

**Published:** 2006-01-05

**Authors:** Fábio Cañellas Moreira, Marcelo Haertel Miglioransa, Marcela Pozo Mautone, Karen Reetz Müller, Fernando Lucchese

**Keywords:** Myocardial diseases, Echocardiography, Differential diagnosis, Cardiomiopatias, Ecocardiografia, Diagnóstico diferencial

## Abstract

Noncompaction of the left ventricular myocardium is a morphogenetic abnormality involving loss of compaction of the myocardial fiber meshwork during intrauterine life. It is an extremely rare condition, accounting for only 0.05% of the cases evaluated in databanks. It has been described in both genders, in many ethnic groups and at different ages. Recently published studies of case series have shown a high mortality rate among these patients during follow-up of up to 48 months. Many cases have so far been misdiagnosed due to poor knowledge of the findings relating to this syndrome. There needs to be an attempt at early and accurate diagnosis, because of the need to investigate the patient's family upon such diagnosis, and today this can be achieved using echocardiographic criteria.

## RESUMO

O miocárdio não compactado do ventrículo esquerdo é uma anormalidade morfogenética que envolve a perda da compactação da trama de fibras do miocárdio durante a vida intra-uterina. É responsável por somente 0,05% dos casos avaliados em bancos de dados. Tem sido descrita em ambos os sexos, em muitos grupos étnicos e em diversas faixas etárias. Estudos de séries de casos publicados recentemente evidenciam a alta mortalidade presente nesses pacientes quando acompanhados por até 48 meses. Talvez, muitos casos até o momento tenham sido mal diagnosticados devido ao pouco conhecimento dos achados da síndrome. O diagnóstico precoce e correto deve ser tentado, e atualmente pode ser feito com critérios ecocardiográficos. A partir do diagnóstico, a investigação familiar se impõe devido aos vários casos de recorrência entre parentes.

## INTRODUCTION

Isolated noncompaction of the left ventricle is a congenital dysfunction of ventricular morphogenesis, considered rare, that occurs because of arrested development of muscle fiber compaction, a process that normally takes place in early embryogenesis.^1^ The arrested morphological and functional development of the heart muscle is characterized by hypertrabeculation and deep recesses in the ventricular wall.^2^

Noncompaction of the myocardium (NCM) was first described by Bellet et al. in 1932 (apud Stöllberger 2004), from an autopsy carried out on a newborn in whom aortic atresia and coronary-ventricular fistula were also observed. Since then, such findings have frequently been reported and described in association with numerous cardiac defects. It was only in 1984 that Engberding et al. reported a case with a diagnosis of isolated (NCM), a condition characterized by the absence of other associated cardiopathies.^3,4^ The clinical aspects of isolated NCM, which can include left ventricular failure, ventricular arrhythmia and thromboembolic events, are similar to those of several types of cardiopathy, thus making clinical differentiation more difficult.^5^ In this respect, echocardiographic findings can provide valuable information. With the increasing number of reported cases, it has become questionable whether this myocardiopathy should really be regarded as rare, since the advances in imaging diagnostic techniques and the recognition of this condition in clinical settings may facilitate its identification.

In the present article, the authors review the existing literature on this topic.

## LITERATURE REVIEW

### Embryology

In the first month of intrauterine life, the myocardium consists of a meshwork of loosely interwoven muscle fibers of spongy appearance. This conformation generates a mesh in which trabeculae alternate with recesses that communicate with the ventricular cavity to provide the blood supply to the cardiac muscle.^2,6^ Between the fifth and eighth weeks of normal development, the ventricular myocardium is gradually compacted, and these recesses turn into capillaries. The process starts from the epicardium and goes towards the endocardium, and from the base to the apex, and it is usually more complete in the left ventricle than in the right ventricle.^7^ Thus, isolated noncompaction syndrome of the left ventricle represents the persistence of multiple trabeculation in the ventricular myocardium (more than three trabeculae) with deep intra- trabecular spaces due to arrested compaction of the wall with no apparent cause or association with other cardiac defects.

### Clinical manifestations

**Figure 1:**
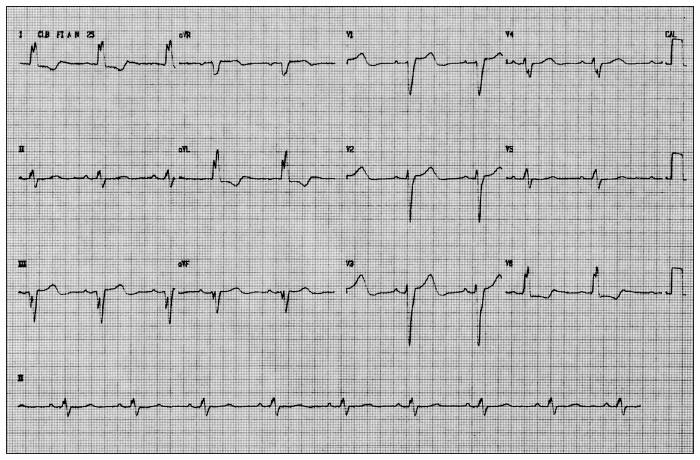
A 12-lead electrocardiogram of a 58-year-old female patient who presented to our institution (Hospital São Francisco, Porto Alegre) with a history of dyspnea during moderate effort, with asthenia and fatigue, accompanied by palpitations, showing a left bundle branch block and a first-degree block.

The clinical aspects of isolated noncompaction of the myocardium are not completely defined yet, as they are not specific to this cardiomyopathy and are highly variable. Patients may be asymptomatic or suffer severe cardiac dysfunction. Among the common manifestations is depression of the systolic function of the left ventricle, which may generate a condition of heart failure. Tachyarrhythmia (atrial fibrillation, supraventricular tachycardia and the Wolff-Parkinson-White syndrome) and ventricular tachycardia, or conduction defects such as atrioventricular or bundle branch block, are also observed (example of a case in Figure 1). Cardioembolic complications, resulting from atrial fibrillation or from the formation of coagula in the ventricular trabeculae, are frequent.^7^

Although it is a congenital cardiomyopathy, the onset of symptoms is widely variable and may occur at an advanced age.^8,9^ Among adult patients, heart failure is more prevalent, while depression of systolic function is more common in pediatric cases.^6^

### Epidemiology

The various studies on isolated noncompaction of the myocardium have shown divergence in the data obtained regarding its prevalence, with values ranging from 0.05% to 0.24% per year.^4^ Moreover, there is also a disparity in these values between different echocardiographic studies, possibly due to variation in the quality of echocardiographic equipment or in the examiners’ professional skills.^4^ Furthermore, the prevalence of isolated NCM may be related to ease of access to imaging tests in certain areas, and to the presence of symptoms that lead the patient to undergo investigation through echocardiography. There is evidence that ethnic characteristics may also be involved, as the disease has several genetic factors.^4^

Among the 223 cases described in one study, 72 patients (32%) were females and 147 (66%) were males, and in four cases (2%) the patient's sex was not informed.^4^ Stöllberger and Finsterer suggested that the higher incidence among males results from differences in the prevalence and severity of myocardial symptoms that lead to greater search for cardiological investigation, or possibly from genetic inheritance patterns.^4^

The disease is ranked in the category of cardiomyopathy types that are not classified by the World Health Organization. With its recognition as a distinct type of cardiomyopathy, the disease will become more widely known in the medical community, thus making it more likely that it will be diagnosed.

### Genetic and familial occurrence

There are no systematic studies on the familial recurrence of NCM. However, there are many reports of its occurrence in several members of the same family (brothers, cousins, parents, children and cases of paternal kin- ship).^4,6^ Although there is male predominance among the reported familial cases, both sexes are affected, both in familial case reports and in non-familial ones.^6^

Two patterns of familial occurrence have been described.^4^ Some families show an X-linked recessive inheritance pattern (as described by Bleyl et al.), while other familial studies suggest heterogeneity in the inheritance pattern.^4,10,11^

The gene responsible for NCM is located in the q28 region of the X chromosome, in the vicinity of other genes that are associated with systemic myopathy (Emery-Dreifuss muscle dystrophy, myotubular myopathy and Barth Syndrome).^6^ Several types of mutations have been described affecting the G4.5 gene, which was initially associated with Barth Syndrome and codes for a family of proteins called ta-fazzin. This gene is also related to X-linked infantile cardiomyopathy, X-linked endocardial fibroelastosis, and isolated NCM.^4,6^ In addition, a mutation has been identified in the gene that codes for α-dystrobrevin or dystrophin as the cause of NCM that is associated with congenital cardiac disease, and in genes responsible for the limited transcription factors for most of the cardiac and mitochondrial mutations. In some cases, however, studies have failed to detect mutations in the G4.5 gene. Another candidate for NCM has been identified in rats: mutation of the FKBP12 gene in rats results in defects in the ventricular septum, dilated cardiomyopathy and NCM. Its human homologue is located in chromosome 20.^6^

The present study allows it to be said only that this is a morphogenetic abnormality with genetic heterogeneity. This is evident, since the appearance of the first reports in families suggested dominant autosomal and recessive autosomal inheritance.^6^

### Diagnosis

**Figure 2:**
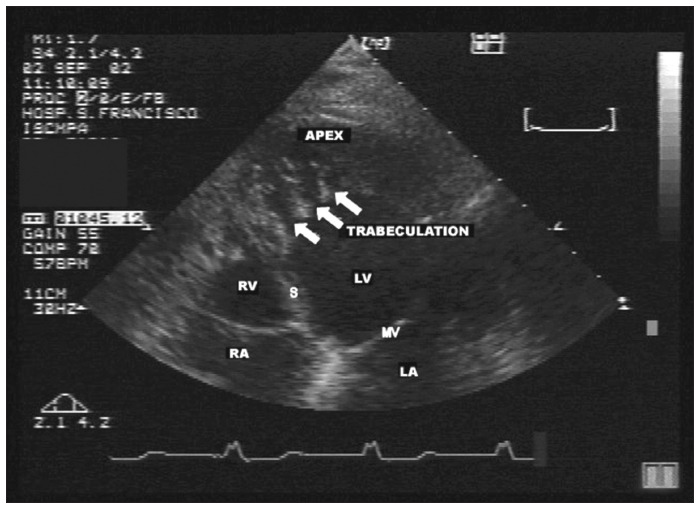
Four-chamber image of the same patient as in Figure 1, showing prominent apical left ventricular trabeculation (white arrow), for whom a diagnosis of noncompaction was suggested. The patient had previously been given an echocardiographic diagnosis of endomyocardial fibrosis.

**Figure 3:**
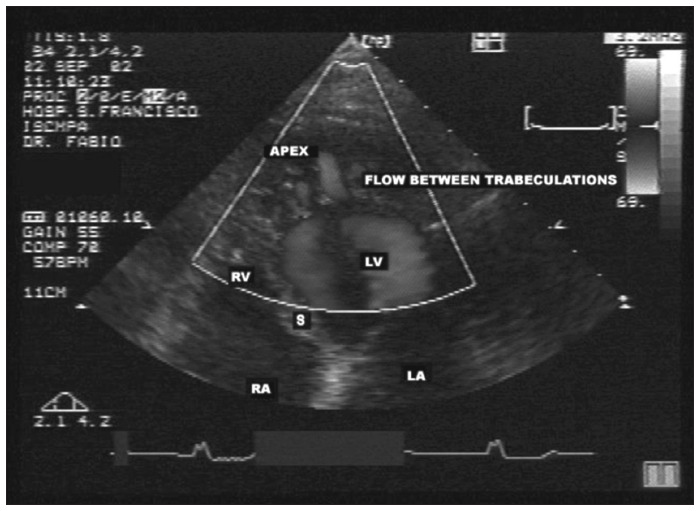
Apical four-chamber view using color Doppler, demonstrating the flow between the prominent trabeculae. This image was obtained from the same patient as in Figures 1 and 2.

The diagnosing of NCM is done by means of bidimensional echocardiography with Doppler.^4^ Chin et al.^12^ suggested that structural alterations in the echocardiogram, especially in the apical region and often in the inferior and lateral segments of the ventricle, are enough for diagnostic confirmation of NCM.^6,12,13^ The disease can be detected by the presence of multiple trabeculation (Figure 2) and deep recesses in communication with the ventricular cavity, as viewed using color Doppler (Figure 3).^14^ All noncompacted segments are hypokinetic but this does not always cause systolic dysfunction, since the disease occurs in dilated as well as hypertrophic or normally-dimensioned ventricles.^6,15^ The morphological findings correspond to what is found in necropsies and in the hearts of patients undergoing transplantation.^16^

*RA = right atrium; RV = right ventricle; S = septum; LA = left atrium; LV = left ventricle; MV = mitral valve.*

*RA = right atrium; RV = right ventricle; S = septum; LA = left atrium; LV = left ventricle.*

**Figure 4:**
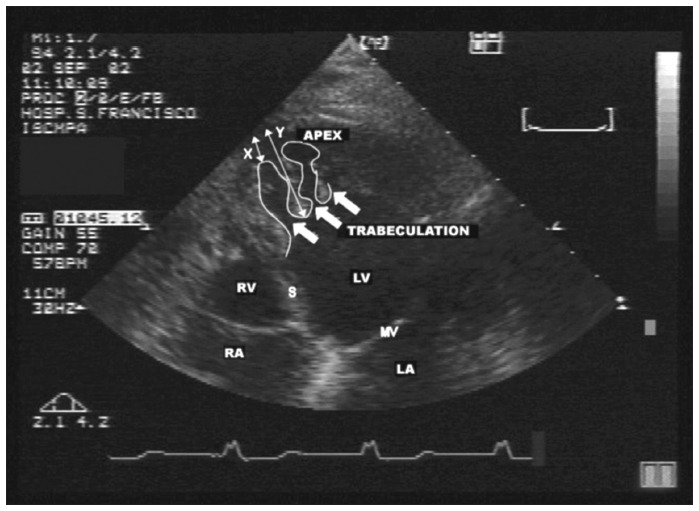
Four-chamber image of the same patient as in Figures 1, 2 and 3, showing the X-to-Y ratio, where X is the distance between the epicardial surface and the recess through and Y refers to the distance between the epicardial surface and the trabecular apex.

The diagnostic criteria for NCM give rise to subjective interpretation, since they depend on good quality of the ultrasonography image, particularly in cases with only small morphological alterations. In order to obtain more accurate diagnosis, Chin et al. introduced a means of quantifying the extent of the trabecular mesh, through calculating the X-to-Y ratio in the diastole.^12^ This ratio is the quotient of the distance between the epicardial surface and the trough of a trabecular recess (represented by X) and the distance between the epicardial surface and maximum trabeculation (represented by Y). An index of greater than or equal to two, from a noncompacted to a compacted zone, is considered to be diagnostic (Figure 4).^7,12^ However, a ratio between the thicknesses of the noncompacted and compacted layers that is greater than two at the end of the systole, as introduced by Oechslin et al., seems to have more clinical applicability.^6,16^

*RA = right atrium; RV = right ventricle; S = septum; LA = left atrium; LV = left ventricle; MV = mitral valve.*

Another group of authors suggested that quantification of the trabeculation can be used as a criterion for defining abnormality, thus suggesting that the disease should be defined by the presence of more than three trabeculae within one image plan, apically to the insertion of the papillary muscles. This criterion can be used even in magnetic resonance and computerized tomography.^4,17^

Computerized tomography and magnetic resonance are useful tools for determining case severity and patients’ prognoses. Through these methods, high-resolution images of the noncompacted myocardium are obtained that enable better recognition of the areas over which the trabeculae are distributed.^18^

The most common abnormalities found on electrocardiograms are the presence of complete or incomplete LBBB (left bundle branch block), possibly together with other conduction disturbances, bradycardia, atrial fibrillation and Wolff-Parkinson-White Syndrome.^6,13^

False interpretations of NCM may occur because of low image quality or, more commonly, because of similarities with other diseases.^4^ The differential diagnosis is made in relation to normal prominent trabeculation of the myocardium; false tendons and anomalous *chordae tendineae*; hypertrophic cardiomyopathy; dilated cardiomyopathy; and thrombi at the ventricular apex.^6,7^ Normal trabeculation is usually located from the free wall of the ventricle up to the septum and is less in number, which thus differentiates it from the multiple apical trabeculae present in NCM.^7,19^ False tendons and anomalous *chordae tendineae* are distinguished from noncompaction of the myocardium because they cross the left ventricular cavity.^6^ In hypertrophic cardiomyopathy, intratrabecular recesses characteristic of NCM are not observed, while in dilated cardiomyopathy there is often some trabeculation in the left ventricle, but of lesser extent than in noncompacted myocardium. Apical thrombi in the left ventricle present different echogenicity in the surrounding myocardium and may give rise to false diagnosis when the noncompaction is restricted to the ventricular apex.^7^

### Prognosis

In recent reports, the prognosis for patients with NCM has been associated with high morbidity and mortality as a result of heart failure, ventricular arrhythmia and systemic embolism.^14^ More specifically, there may be differences between adult and pediatric patients, with systemic embolism being more common among adults.^6^

The main cause of death appears to be arrhythmia resulting from the NCM, which can lead to sudden death. Premature heart transplantation or implantation of a defibrillator may be necessary in some cases.^6^

The population selected in these studies, however, may correspond to a group of mainly symptomatic patients who had to seek medical care, which would thus contribute towards the bad prognoses in most of these reports. At present, it is known that cases with good myocardial function and absence of arrhythmia usually present good disease course, possibly without symptoms for a long time, or there may be development of cardiac failure with good response to drug therapy.^4,13^

### Treatment

There is no specific therapy for NCM. Thus, treatment is restricted to alleviation of the symptoms and complications that the patient may present. The manifestations of heart failure can be treated with the usual drugs for this, which include beta-blockers, angiotensin-converting enzyme inhibitors and diuretics.^4^ In the cases in which such therapy is not successful, an indication for heart transplantation may be considered.^13^

Due to the high incidence of thromboembolic events in patients with NCM, systemic anticoagulation of all patients with this malformation is indicated.^13^

For arrhythmia, whether symptomatic or not, administration of anti-arrhythmic drugs is primarily indicated. However, in some patients, the implantation of a cardioverter-defibrillator may be necessary, depending on the severity and repercussion of these events.^4^

### Association with other diseases

Noncompaction of the left ventricular myocardium has been associated with other congenital cardiac malformations, such as abnormalities of the origin of the left coronary artery starting from the trunk of the pulmonary artery; pulmonary atresia and stenosis; obstruction of the right or left ventricle outflow tract; defects in the ventricular and atrial septum; and hypoplastic left ventricle.^19^

Cases of NCM associated with mitral regurgitation have been reported as well. In these situations, the leaves of the mitral valve become thickened, leading to restriction of their movement and incomplete closure of the valve. At first this mitral defect was thought to be due to systolic dysfunction of the left ventricle, but this hypothesis was dismissed after the description of cases in which left ventricular function was preserved.^20^

The alterations associated with NCM are not restricted to cardiac defects. Some neuromuscular disorders may also appear, such as Becker's muscular dystrophy, mitochondrial myopathy, polyneuropathy and metabolic myopathy, among others.^4^

Some facial malformations may also be associated. There is a certain pattern in these disorders, which include strabismus, low insertion of the ears, prominent forehead, elevated palate arch, saddle nose, and micrognathia.^21^

Both the isolated and the non-isolated forms of NCM are associated with other malformations that also result from mutation of the G-4.5 gene. Some of these malformations are Emery-Dreifuss muscular dystrophy, myotubular cardiomyopathy and Barth Syndrome. The latter is characterized by the presence of skeletal myopathy, delayed growth, neutropenia, lactic acidosis, abnormal cholesterol metabolism, increased levels of organic acids in the urine, decreased concentration of carnitine and mitochondrial anomalies.^21^

There are cases of isolated NCM associated with the presence of Melnick-Needles Syndrome, which comprises unusual bone modeling that is characterized by facial dysmorphism and, occasionally, multiple anomalies.^13^

## CONCLUSION

Isolated NCM is a rare congenital malformation of variable frequency and wide spectrum of clinical manifestations. The increasing numbers of reported cases have brought greater prominence for this entity among the scientific community. There is evidence of familial recurrence and this is perhaps the most important factor in its differential diagnosis, since the treatment is, for cases with ventricular dysfunction, the same as is prescribed for patients suffering from heart failure. Distinct morphologic characteristics and functional repercussions can be diagnosed by means of bidimensional echocardiograms, which are the main screening method. However, because of the few cases described in the literature and consequently the limited awareness that some echocardiographers have with this disease, clinicians must include this condition in the differential diagnosis and express such suspicions.

## References

[1] Grillo R, Pipitone S, Mongiovi M (2002). Non compattazione isolata del ventricolo sinistro in età pediatrica: esperienza clinica su cinque casi. [Isolated non-compaction of left ventricle in childhood: clinical experience with 5 cases]. Ital Heart J Suppl.

[2] Zambrano E, Marshalko SJ, Jaffe CC, Hui P (2002). Isolated noncompaction of the ventricular myocardium: clinical and molecular aspects of a rare cardiomyopathy. Lab Invest.

[3] Engberding R, Bender F (1984). Identification of a rare congenital anomaly of the myocardium by two-dimensional echocardiography: persistence of isolated myocardial sinusoids. Am J Cardiol.

[4] Stöllberger C, Finsterer J (2004). Left ventricular hypertrabeculation/noncompaction. J Am Soc Echocardiogr.

[5] Buonanno C, Variola A, Dander B, Gabalso S, Marafioti V (2000). Isolated noncompaction of the myocardium: an exceedingly rare cardiomyopathy. A case report. Ital Heart J.

[6] Rigopoulos A, Rizos IK, Aggeli C (2002). Isolated left ventricular noncompaction: an unclassified cardiomyopathy with severe prognosis in adults. Cardiology.

[7] Corrado G, Santarone M, Miglierina E (2000). Isolated noncompaction of the ventricular myocardium. A study in an adult male and literature review. Ital Heart J.

[8] Takashima A, Shimizu M, Tatsumi K, Shima T, Miwa Y (2004). [Isolated left ventricular noncompaction in the elderly: a case report]. J Cardiol.

[9] Lin ML, Connelly K, Prior D (2005). An unusual cause of heart failure identified by echocardiography in an octogenarian. Eur J Heart Fail.

[10] Bleyl SB, Mumford BR, Brown-Harrison MC (1997). Xq28-linked noncompaction of the left ventricular myocardium: prenatal diagnosis and pathologic analysis of affected individuals. Am J Med Genet.

[11] Bleyl SB, Mumford BR, Thompson V (1997). Neonatal, lethal noncompaction of the left ventricular myocardium is allelic with Barth syndrome. Am J Hum Genet.

[12] Chin TK, Perloff JK, Williams RG, Jue K, Mohrmann R (1990). Isolated noncompaction of left ventricular myocardium. A study of eight cases. Circulation.

[13] Elias J, Valadão W, Kuniyoshi R, Queiroz A, Peixoto CA (2000). [Isolated noncompaction of the myocardium]. Arq Bras Cardiol.

[14] Williams RI, Masani ND, Buchalter MB, Fraser AG (2003). Abnormal myocardial strain rate in noncompaction of the left ventricle. J Am Soc Echocardiogr.

[15] Stöllberger C, Finsterer J (2005). Cardiologic and neurologic findings in left ventricular hypertrabeculation/non-compaction related to wall thickness, size and systolic function. Eur J Heart Fail.

[16] Oechslin EN, Attenhofer Jost CH, Rojas JR, Kaufmann PA, Jenni R (2000). Long-term follow-up of 34 adults with isolated left ventricular noncompaction: a distinct cardiomyopathy with poor prognosis. J Am Coll Cardiol.

[17] Stöllberger C, Finsterer J, Blazek G (2002). Left ventricular hypertra-beculation/noncompaction and association with additional cardiac abnormalities and neuromuscular disorders. Am J Cardiol.

[18] Hamamichi Y, Ichida F, Hashimoto I (2001). Isolated noncompaction of the ventricular myocardium: ultrafast computed tomography and magnetic resonance imaging. Int J Cardiovasc Imaging.

[19] Ozkutlu S, Ayabakan C, Celiker A, Elshershari H (2002). Noncompaction of ventricular myocardium: a study of twelve patients. J Am Soc Echocardiogr.

[20] Ali SK, Omran AS, Najm H, Godman MJ (2004). Noncompaction of the ventricular myocardium associated with mitral regurgitation and preserved ventricular systolic function. J Am Soc Echocardiogr.

[21] Siles Rubio JR, Arizon Del Prado JM, Lopez Granados A, Mesa Rubio D, Lopez Rubio F, Ramirez Moreno A (2002). [Isolated form of spongy myocardiopathy]. Rev Esp Cardiol.

